# Inhibition of IL-27 signaling regulates chemokine levels and sustains CXCR2 receptor expression on mononuclear cells to improve disease outcomes during gram-negative neonatal sepsis

**DOI:** 10.3389/fimmu.2025.1653355

**Published:** 2025-09-05

**Authors:** Madhavi Annamanedi, Jordan K. Vance, Cory M. Robinson

**Affiliations:** ^1^ Department of Microbiology, Immunology, & Cell Biology, West Virginia University School of Medicine, Morgantown, WV, United States; ^2^ Vaccine Development Center, West Virginia University Health Sciences Center, Morgantown, WV, United States

**Keywords:** CXCR2, CXCL2, monocytes, chemokine, interleukin-27, neonatal sepsis, bacterial infection, bacterial clearance

## Abstract

**Background:**

Interleukin-27 (IL-27) is a cytokine that belongs to the IL-6/IL-12 cytokine family with diverse influences on the immune response. Elevated levels of IL-27 cytokine during the neonatal period predispose neonatal mice to more severe infection. Neonatal pups deficient in IL-27 signaling exhibit improved survival and bacterial clearance with reduced systemic inflammation. However, the precise molecular mechanisms that regulate bacterial clearance and the overall immune response in IL-27 receptor a-deficient (KO) mice during neonatal sepsis remain incompletely defined.

**Methods:**

Analysis of the transcriptome of the neonatal spleen during *Escherichia coli*-induced sepsis in IL-27Rα KO mice identified elevated expression of the chemokine receptor gene CXCR2. Here we further explored the mechanistic insights of the CXCR2/CXCL2 signaling axis limiting the infection in WT and IL-27Rα KO neonatal mice using an *n vivo* model and *ex vivo* studies with primary cells.

**Results:**

The results uncovered that during infection WT neonatal mice fail to increase expression of CXCR2 but upregulate the cognate ligand CXCL2 significantly. Conversely, IL-27Rα KO neonates increase CXCR2 expression significantly in the spleen during infection but fail to upregulate CXCL2 transcripts. Splenocytes isolated form septic neonatal KO mice migrated with superior efficiency towards the chemokine CXCL2 compared to WT counterparts. Surprisingly, we also found that splenic monocytes but not the neutrophils account for higher CXCR2 gene expression in the IL-27Rα KO neonatal mice. Monocytes isolated from the spleens of both WT and IL-27Rα KO neonatal pups confirmed that the concentration of CXCL2 regulates CXCR2 receptor expression. We further demonstrated that with regulated CXCL2 chemokine expression levels, IL-27Ra-deficient neonatal mice had more CXCR2+ mononuclear cells present at the site of infection.

**Conclusions:**

Overall, our findings suggest that during infection in the absence of IL-27 signaling, a differential expression of CXCR2 and CXCL2 promotes increased migration of mononuclear cells consistent with improved bacterial clearance and tissue homeostasis. This study defines mechanisms that improve the host response in the absence of IL-27 signaling during neonatal sepsis and reinforces the potential for antagonizing IL-27 as a host-directed therapy for neonatal sepsis.

## Introduction

Neonatal sepsis is a dysregulated host response due to infection in the blood which occurs first 28 days of life that is life-threatening with a high rate of mortality (1). It is responsible for approximately 8% of all neonatal deaths and not only represents the leading cause of neonatal mortality but also contributes to long-term morbidity (2). Neonatal sepsis is categorized into early onset (0-3 day of life) and late onset (>3 days of life). In early-onset sepsis neonates most commonly acquire infectious agents from the mother via ascension from the cervix or from the colonized birth canal at the time of delivery. Group B streptococci and *E. coli* are the most common aetiologic bacteria of early onset neonatal sepsis (3). Late onset sepsis is common in extremely low birth weight premature infants who experience longer durations in the hospital (4). Hospital-acquired pathogens from intensive care such as gram-positive bacteria, coagulase negative staphylococci (CoNS), and gram-negative bacterial species are commonly associated with late onset of sepsis (5). Treatment for neonatal sepsis is limited to a combination of antibiotics and supportive care (6, 7). First-line treatment for neonatal sepsis is a beta-lactam antibiotic such as ampicillin combined with an aminoglycoside that is generally gentamicin. Cefotaxime or vancomycin are primary option to treat late onset sepsis (8). Difficulties or delayed diagnosis, selecting the wrong antibiotics, and emergence of antibiotic resistance are major treatment challenges of neonatal sepsis (9). Several adjuvant therapies have been explored with intravenous immunoglobulin administration among the well-studied, but these approaches have failed to change the prognosis for this serious infant disease (10). The neonatal immune system is not fully developed in the transition from maternal tolerance and is inefficient in the clearance of microbial pathogens (11). Neonates rely heavily on innate immunity that is polarized toward an anti-inflammatory state (12). A detailed understanding of mechanisms that can augment and improve innate immune responsiveness in neonates may facilitate the development of new therapeutic strategies to treat neonatal sepsis (11).

Interleukin (IL)-27 is a heterodimer composed of IL-27p28 and Epstein-Barr virus–induced 3 (EBI3) subunits that signal through a cell surface receptor composed of IL-27Rα and gp130. IL-27 promotes Th1, Th2 and Treg cell differentiation and inhibits Th17 cell differentiation (13–16). IL-27 is primarily produced by antigen-presenting cells such as dendritic cells, macrophages, monocytes, and more recently, B cells (17, 18). IL-27 is known to play a role in the immune response during sepsis; serum levels are elevated during the neonatal period and increase further upon infection (19–22). At the time of infection IL-27 promotes bacterial persistence and compromises host defense (20, 23). Loss of IL-27 signaling improves the survival rate in an experimental murine neonatal sepsis model (20). In a mouse model of cecal ligation and puncture (CLP)-induced acute lung injury, IL-27 neutralization lowered pulmonary inflammation and enhanced survival rate (24). Blockade of IL-27 signaling in a murine model of secondary *Staphylococcus aureus* pneumonia also improved bacterial clearance (23). However, understanding the precise role of IL-27 during bacterial infections early in life remains incomplete. Neonatal sepsis studies from our group using IL-27Rα-deficient (KO) mice, revealed that absence of IL-27 signaling improved maintenance of body mass, increased bacterial clearance with reduced systemic inflammation, and decreased mortality rates in the pups (20). Our laboratory studied the transcriptome of the neonatal spleen during *Escherichia coli*-induced sepsis in wild-type (WT) and IL-27Rα-deficient (KO) mice (25). Among the important findings in this work are a significant increase in expression of cytokines and chemokines during infection in WT pups that failed to upregulate in the KO pups, consistent with overall lower levels of inflammation and improved outcomes (25). However, in contrast to this trend, we identified elevated levels of the chemokine receptor gene, CXCR2, in the KO neonatal mice during infection that did not change significantly in WT pups (25).

CXCR2 is found on many cells including leukocytes, endothelial, and epithelial cells and is a high-affinity receptor for IL-8 in humans and CXCL2 in mice (26). In sepsis, the chemokine CXCL2 and its receptor CXCR2 play a critical role in neutrophil and monocyte recruitment and promotes their migration to inflammatory sites (27, 28). Here, we explored the mechanism by which elevated levels of CXCR2 aid in combating sepsis in IL-27Rα KO neonatal mice.

## Materials and methods

### Ethics statement

All procedures were approved by the West Virginia University Institutional Animal Care and Use Committee (Protocol #:1708008935) and conducted in accordance with the recommendations from the Guide for the Care and Use of Laboratory Animals by the National Research Council (NRC, 2011).

### Mice

Breeding pairs of C57BL/6 (WT) or IL-27Rα^-/-^ (KO) mice on a C57BL/6 genetic background were purchased from Jackson Laboratories (Bar Harbor, ME) and maintained under specific pathogen-free conditions in the vivarium at West Virginia University Health Sciences Center. Mice were maintained on a 12-h light/dark cycle and were fed/watered ad libitum. Male and female pups (~300) were used for experimental infection. Blood and tissues were collected from mice at the appropriate age by humane procedures. Euthanasia procedures involved isoflurane inhalation at 4-5% induction.

### Bacterial infection of neonatal mice

*Escherichia coli* isolates with serotype O1 are invasive and frequently express the K1 capsule, a virulence factor associated to neonatal meningitis, bacteremia, and septicemia (29). We established a murine neonatal sepsis model using *Escherichia coli* strain O1:K1:H7 (ATCC 11775) obtained from the ATCC (Manassis, VA, USA). The bacteria were grown in Luria broth from a single colony isolated on Tryptic Soy agar (TSA). To prepare infectious inoculums, the bacteria were enumerated as described previously (21). Neonatal pups (n=3 per group [control vs. infected] per genotype [WT vs. KO]) at 4 days were inoculated subcutaneously in the scapular region with *E. coli* O1:K1:H7 using a 28-gauge insulin needle as described previously (20, 21). The bacteria were washed with PBS, centrifuged at 2,000 x g for 5 min, and suspended in a volume of PBS equivalent to an inoculum of 50 μL/mouse. The target inoculum was 2×10^5^ CFUs per mouse and actual inoculums as determined by standard plate counts, ranged from 2-3x10^5^ CFUs per mouse. Vehicle (PBS)-received pups were identified from *E. coli*-infected pups using a tail snip. The weights of mice were recorded immediately prior to infection, and at 24 h immediately prior to euthanasia. Blood glucose levels were measured using an AlphaTrack3 blood glucose monitoring system (Zoetis, MI, USA). All downstream experiments were associated with the same pre-identified mice. Spleens isolated from pups were placed in PBS on ice. For some experiments, spleens were placed in 4% paraformaldehyde for further histopathological studies. Blood was deposited in tubes that contained 5 μL of 500 mM ethylenediamine tetraacetate acid (EDTA) and placed on ice. Serum was collected by centrifuging at 2,000 x g for 10 min. The bacterial burden in the spleen was enumerated by serial dilution and standard plating on TSA.

### RNA isolation and quantitative real time PCR

Spleens were homogenized in TRI Reagent^®^ (Molecular Research Center, Cincinnati, OH). According to the commercial product protocol, the upper aqueous layer following phase separation was mixed with an equal volume of 75% ethanol and transferred to E.Z.N.A.^®^ RNA isolation columns (Omega Biotek, GA, USA). The manufacturer’s instructions were followed to complete tissue RNA isolation using E.Z.N.A.^®^ RNA isolation kit (Omega Biotek, GA, USA). RNA from cells or tissue was quantified using a nanodrop. First-strand cDNA was synthesized using the iScript™ cDNA synthesis kit (Bio-Rad, CA, USA). Quantitative PCR reactions included cDNA diluted four-fold, gene-specific TaqMan^®^ primer probe sets (ThermoFisher, MA, USA), and iQ™ Supermix (Bio-Rad, CA, USA). Cycling was performed in triplicate using a StepOnePlus™ Real-Time detection system (ThermoFisher, MA, USA). Gene-specific amplification as an internal reference gene and expressed as log_2_ relative gene expression compared to control spleen using the formula 2^-ΔΔCt^. CXCR2 (ID: Mm. PT. 58. 12102629) and CXCL2 (ID: Mm. PT. 58.10456839) assays were purchased from Integrated DNA Technologies (Coralville, IA, USA). The β-actin assay (ID: mM01205647_g1) was purchased from ThermoFisher (MA, USA).

### Chemotaxis assay

Splenocytes (5x10^5^) from infected WT and KO animals isolated as described above were added onto the membrane of the 24-transwell insert (3 μm pore size) in a volume of 100 µL DMEM. A volume of 600 µL complete DMEM with or without 0.025 ng/mL CXCL2 chemoattractant was carefully added to the well. The transwell culture was incubated at 37°C with 5% CO_2_. Images of migrated cells in the medium of the bottom chamber were taken at 1 and 4 h post-incubation using an Evos imaging system (Thermo Fisher Scientific, Massachusetts, USA). Average of 4 picture fields at 10x magnification were taken for each condition and cell counts were determined using ImageJ automated software (30).

### Cell separation

Four-day old WT (n=5) and KO (n=5) pups infected for 24 h were humanely euthanized and the spleens collected. To obtain sufficient cells for downstream analysis, spleens were pooled based on infection status (control vs. infected). Single-cell suspensions were generated by dissociating the spleens through a 40 µm strainer and centrifugation at 350 × g for 5 min. Cells were resuspended in 0.5 mL of ammonium/chloride/potassium erythrocytes lysis buffer (ThermoFisher Scientific, Massachusetts, USA) and incubated on ice for 5 min, followed by centrifugation at 350 × g for 5 min. Mononuclear leukocytes were enriched from splenocytes using Optiprep™ (Sigma-Aldrich, Illinois, USA) density gradient centrifugation as described previously and scaled down four-fold to accommodate the smaller number of cells in neonatal spleens (21, 31). Neutrophils were isolated from splenocytes with magnetically labeled anti-Ly6B.2 (7/4) microbeads and immunomagnetic selection using Miltenyi Biotec isolation reagents (Miltenyi Biotec, Bergisch Gladbach, Germany) as described previously (20, 32) Splenocytes, monocytes and neutrophils were counted using automated cell countess 3 (Invitrogen, Massachusetts, USA) used as described in downstream approaches. The enrichment of mononuclear cells and neutrophils was assessed by immunolabeling for CD11b, Ly6C, and Ly6G, respectively.

### Ex-vivo studies of CXCR2 expression

Mononuclear cells from WT and KO pups of seven day old were isolated as described above and 1×10^6^ cells seeded per well in a 6 well plate. These cells were treated with CXCL2 0.1µg/mL or 1µg/mL for 8 h. Cells were harvested, and RNA was extracted to check the gene expression of CXCR2.

### Flow cytometry

5×10^5^ cells splenocytes or mononuclear cells were mixed with Fc receptor blocking reagent (Miltenyi Biotec, Bergisch Gladbach, Germany) and held at room temperature for 10 min prior to cell surface marker labeling with fluorochrome-conjugated antibodies. In addition to Live/Dead stain (FVS780, BD Biosciences, CA, USA), antibodies used for the panel included CD11b (BV786) and Ly6C (PE) from BD Biosciences and CXCR2 (APC) from Miltenyi Biotec. All antibodies were diluted according to manufacturer specifications. Immunolabeling was performed for 45 min on ice protected from light. Cells were washed, resuspended in 4% paraformaldehyde (PFA; ThermoFisher Scientific, MA, USA), and stored at 4 °C until flow cytometric analysis. All samples were analyzed on a LSRFortessa instrument (BD Biosciences) using FCS Express version 7.0 software. A minimum of 30,000 events were analyzed per sample.

### Pathological assessments

Spleens were fixed in 4% paraformaldehyde, embedded in paraffin, sectioned at a thickness of 3 μm, and then stained with hematoxylin & eosin (H&E) solution according to standard procedures performed by the Electron Microscopy Histopathology and Tissue Bank Core Facility at West Virginia University. Histopathologic changes were observed by light microscopy (Olympus slide scanner, Japan). Organ damage was evaluated by measuring aspartate aminotransferase (Cayman, MI, USA) in the serum according to manufacturer’s protocol.

### Statistical analysis

All data sets were analyzed with the appropriate parametric or nonparametric test as indicated in the figure legend using Prism 8 (GraphPad, San Diego, CA). The threshold for statistical significance was set at alpha = 0.05.

## Results

### IL-27 influences expression levels of CXCR2 in the spleen during neonatal sepsis

**Figure 1:**
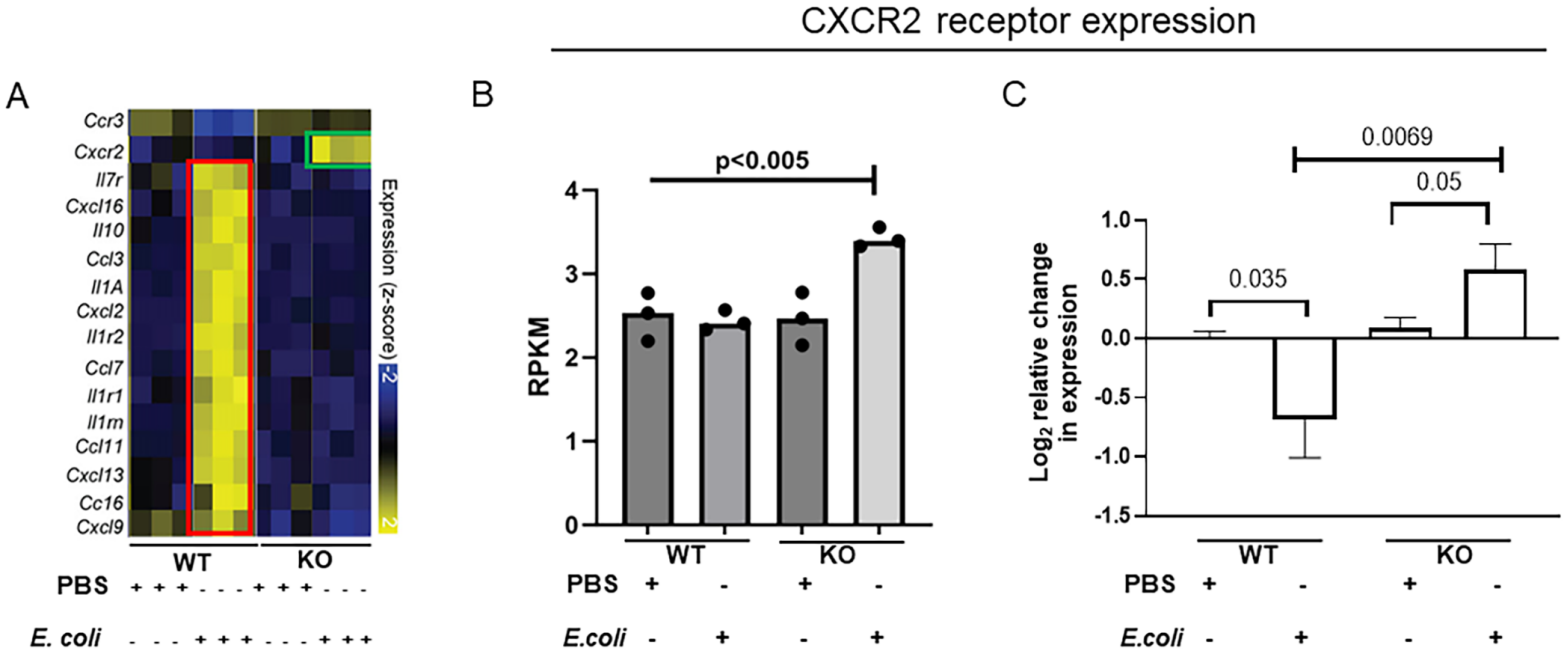
IL-27Rα KO neonatal mice increase CXCR2 chemokine receptor expression during *E. coli*-induced sepsis. Neonatal WT and KO mice (n=3-4) were subcutaneously inoculated with a target of 2×10^5^ CFU/mouse of *E. coli* O1:K1:H7 or PBS as a control on day 4 of life. Spleens were collected at 24 h post-infection. **(A)** Heatmap visualization of expression values for a subset of genes annotated as chemokine receptors, interleukins, and chemokine ligands that are differentially expressed between WT and KO spleens in the presence or absence of *E. coli* infection from our previously published data (25). Highlighted changes in KO pups (green framed) are contrasted with those in WT pups (red framed). **(B)** The relative molar concentration (rmc) of a transcript in a RNA sample described previously (25), was measured and represented in reads per kilobase million values (RPKM). **(C)** Mean gene expression levels of CXCR2 ± standard error (SE) in the spleen is shown for 3 independent experiments. The expression was determined relative to uninfected control spleens by real-time PCR using the formula 2^-ΔΔCt^. Statistical significance in the 95% confidence interval was determined using individual unpaired two tailed t tests; exact P values are shown.

To understand the impact of IL-27 on regulation of the neonatal host response to infection, we previously analyzed the transcriptome of the neonatal spleen during K1-encapsulated *E. coli*-induced sepsis in WT and KO mice (25). KO mice do not express a functional receptor and cannot respond to IL-27. Many genes for cytokines, chemokines, and their receptors were increased in WT animals during infection, consistent with pronounced inflammation, but did not increase significantly in KO pups (**Figure 1A**) (25). In contrast, CXCR2 expression was upregulated significantly in KO neonates during infection but failed to increase in WT neonates (**Figures 1A, B**) (25). To further confirm these findings in new sets of animals, WT and KO neonates were infected on day 4 with *E. coli* and the spleens collected at 24 h post-infection to determine CXCR2 gene expression. CXCR2 expression levels in the KO neonates were significantly higher during infection (**Figure 1C**). WT neonates downregulated CXCR2 expression levels in the spleen upon *E. coli* infection (**Figure 1C**).

### CXCL2 is differentially expressed in the absence of IL-27 signaling during neonatal infection

**Figure 2:**
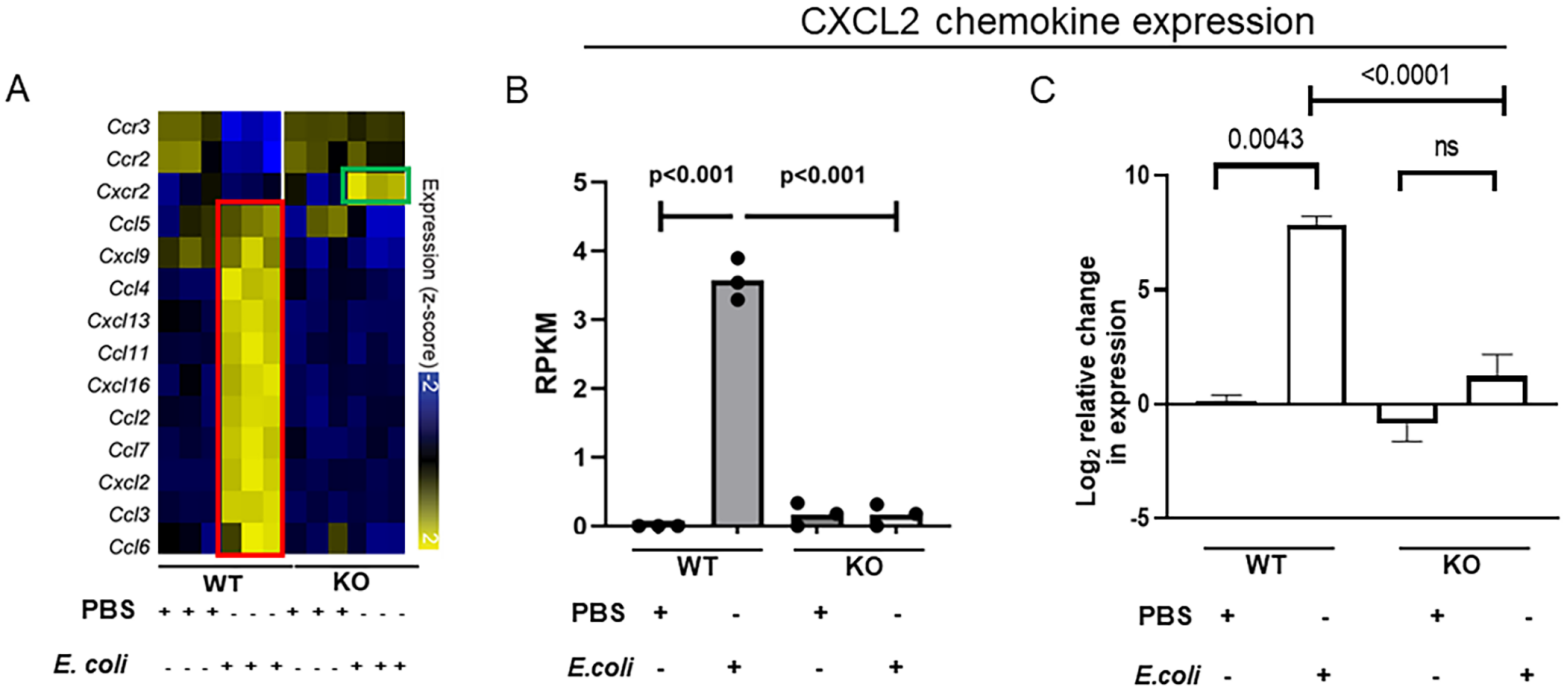
WT neonatal mice upregulate CXCL2 chemokine expression significantly during *E. coli*-induced sepsis. Neonatal WT and KO mice (n=3-4) were subcutaneously inoculated with a target of 2×10^5^ CFU/mouse of *E. coli* O1:K1:H7 or PBS as a control on day 4 of life. Spleens were collected at 24 h post-infection. **(A)** Heatmap visualization of expression values for a subset of genes annotated as chemokine receptors, and chemokine ligands that are differentially expressed between WT (red framed) and KO (green framed) samples in the presence or absence of *E. coli* infection. **(B)** The relative molar concentration (rmc) of a transcript in a RNA sample described previously (25), was measured and represented in reads per kilobase million values (RPKM). **(C)** Mean gene expression levels of CXCL2 ± SE in the spleen are shown for 3 independent experiments. The expression was determined relative to uninfected control spleens by real-time PCR using the formula 2^-ΔΔCt^. Statistical significance in the 95% confidence interval was determined using individual unpaired two tailed t tests; exact P values are shown.

Transcriptome data showed that WT neonates had very high expression levels of numerous chemokines during infection (**Figure 2A**) (25). Among them CXCL2 is one of the high- affinity binding ligands for CXCR2 receptor (33). Gene expression data from the spleens of WT and KO neonates confirmed that during infection, CXCL2 chemokine levels were highly elevated in the WT (**Figures 2B, C**) whereas KO had moderately elevated expression (**Figure 2C**). In contrast to the higher levels of chemokine CXCL2 levels, the receptor expression is very low in the WT infected pups (**Figures 1**, **2**). In the spleen, although IL-27 receptor KO infected pups had moderate expression levels of chemokine CXCL2, its receptor CXCR2 levels were high (**Figures 1**, **2**).

### The absence of IL-27 signaling promotes effective migration of splenocytes towards chemoattractant

**Figure 3:**
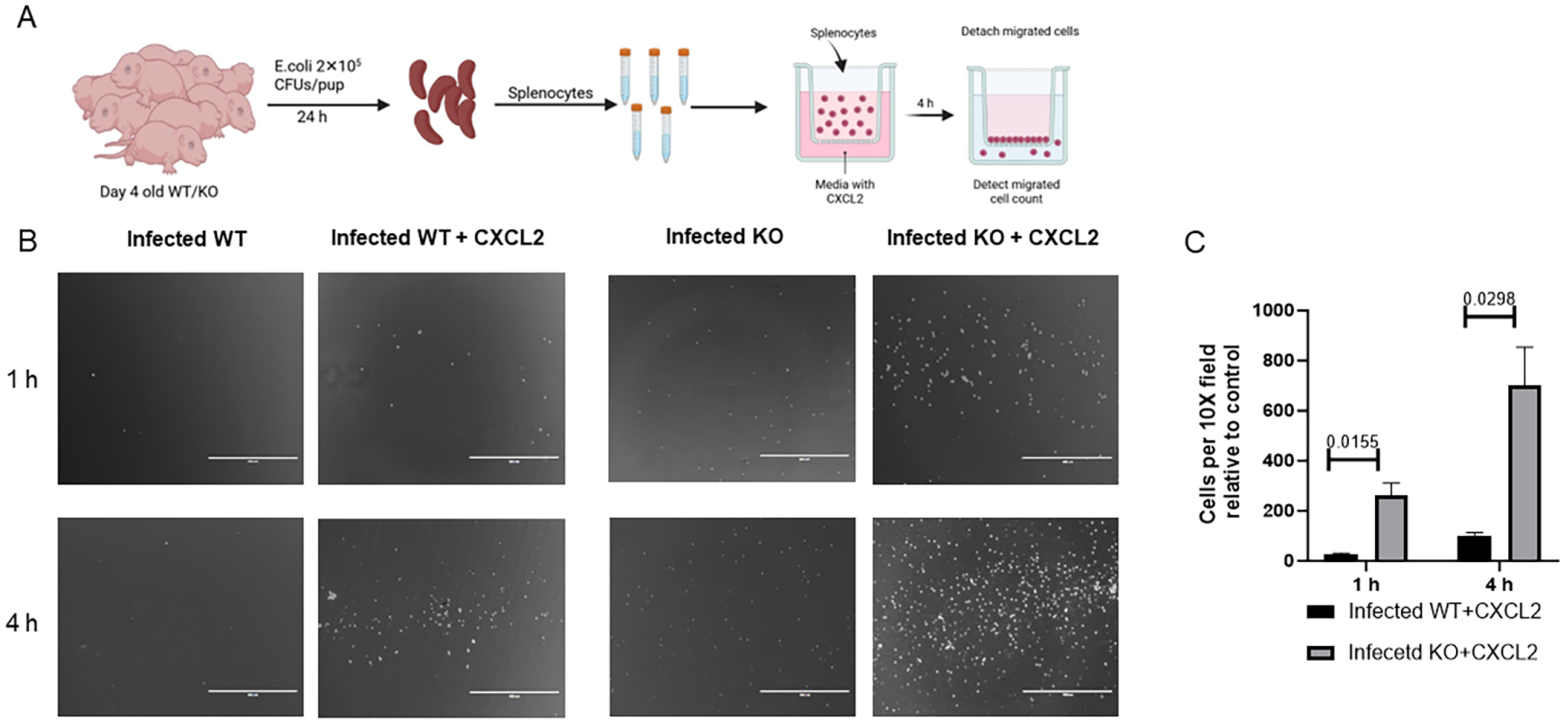
Efficient migration of neonatal splenocytes from infected IL-27Rα KO pups towards chemoattractant. Splenocytes were prepared at 24 h post-infection from the spleens of neonatal mice infected with 2×10^5^ CFUs of *E coli* O1:K1:H7 (n=4-5) on day 4 of life. The cells were placed in a 3 μm pore size trans-well insert with complete media ± CXCL2 placed in the bottom chamber. Migrated cells were imaged and counted at 1 and 4 h post-incubation. **(A)**. Schematic representation of the methodology. **(B)**. Representative images of migrated cells at the indicated time point and condition; scale bar is 400µm. **(C)**. Quantification of WT and KO cells migrating towards the chemo-attractant in relative to control (without CXCL2) for 1 and 4 h Data shown was obtained from at least 4 picture fields at 10x total magnification per experiment from a combined 3 independent experiments. Statistical significance was determined using individual unpaired two tailed t tests; exact P values are shown.

Chemotaxis of myeloid cells from the blood and bone marrow is an important process to coordinate immune responses within the spleen, enabling host response to infection and maintenance of tissue homeostasis (34). This chemotaxis is regulated by various factors, including chemokine receptors on cells and chemokines expressed by cells at the target tissues (35). To determine if increased CXCR2 expression on KO splenocytes promoted enhanced migration, we measured chemotactic migration toward CXCL2 using an *in vitro* Boyden chamber assay (**Figure 3A**). WT and KO neonatal pups were infected with *E. coli* and spleens were harvested at 24 h post-infection (**Figure 3A**). Splenocytes were isolated, placed in the top of the transwell, and cells migrated toward CXCL2 in the bottom well were measured by microscopy. The results demonstrated that infected KO neonatal splenocytes exhibited greater migration efficiency compared to infected WT neonatal cells over 1-4 h (**Figures 3B, C**).

### CXCR2 gene expression is selectively increased in the mononuclear cell population of the spleen in the absence of IL-27 signaling during infection

**Figure 4:**
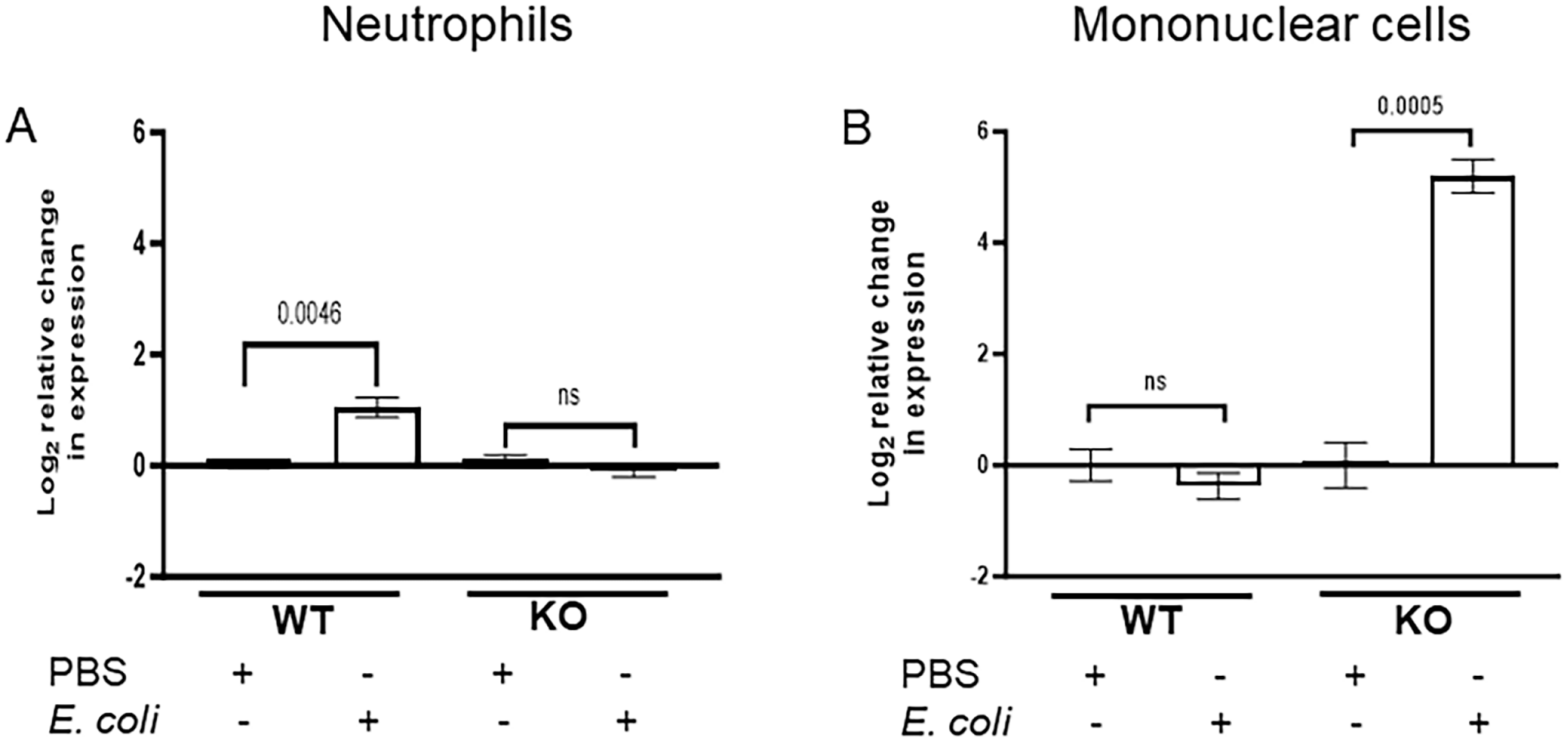
CXCR2 expression is selectively increased in splenic mononuclear cells during neonatal infection in the absence of IL-27 signaling. Neonatal WT and KO mice were subcutaneously inoculated with a target inoculum of 2×10^5^ CFU/mouse of *E. coli* O1:K1:H7 (n=5) or PBS (n=5) as a control on day 4 of life. Spleens were collected at 24 h post-infection for enrichment of neutrophils and mononuclear cells. Mean gene expression levels of CXCR2 ± SE in **(A)** neutrophils or **(B)** mononuclear cells from a combined 3 independent experiments. The expression was determined relative to uninfected control spleens by real-time PCR using the formula 2^-ΔΔCt^. Statistical significance was determined using individual unpaired two tailed t tests; exact P values are shown.

CXCR2 is expressed by mixed phagocytic myeloid cells such as neutrophils, monocytes, and macrophages and mediates migration of inflammatory cells (36, 37). The role of CXCR2 in neutrophil recruitment to inflammatory sites has been well established and shown to control the magnitude of the macrophage-dependent inflammatory response (38, 39). It is also known to regulate monocyte recruitment and function (40). After confirming elevated levels of CXCR2 and migration efficiency of KO splenocytes infected neonatal mice, we investigated the specific cell type influencing CXCR2 expression levels in the spleen. As such, we isolated neutrophils and mononuclear cells from the spleens of control and infected WT and KO neonatal mice. Anti-Ly6B2 magnetic beads were used to enrich neutrophils, and monocytes were isolated by density gradient centrifugation (41, 42). We observed an approximate two-fold increase in CXCR2 expression in splenic neutrophils from WT pups during infection relative to uninfected controls (**Figure 4A**). In contrast, there was no change in CXCR2 expression during infection in neutrophils from KO pups (**Figure 4A**). Surprisingly, there was no significant change in the CXCR2 expression observed in splenic mononuclear cells from WT pups during infection (**Figure 4B**). However, a dramatic 32-fold increase of CXCR2 expression levels observed in the mononuclear cells of infected KO neonates relative to uninfected controls (**Figure 4B**).

### Chemokine CXCL2 levels regulate CXCR2 receptor expression on neonatal mononuclear cells

**Figure 5:**
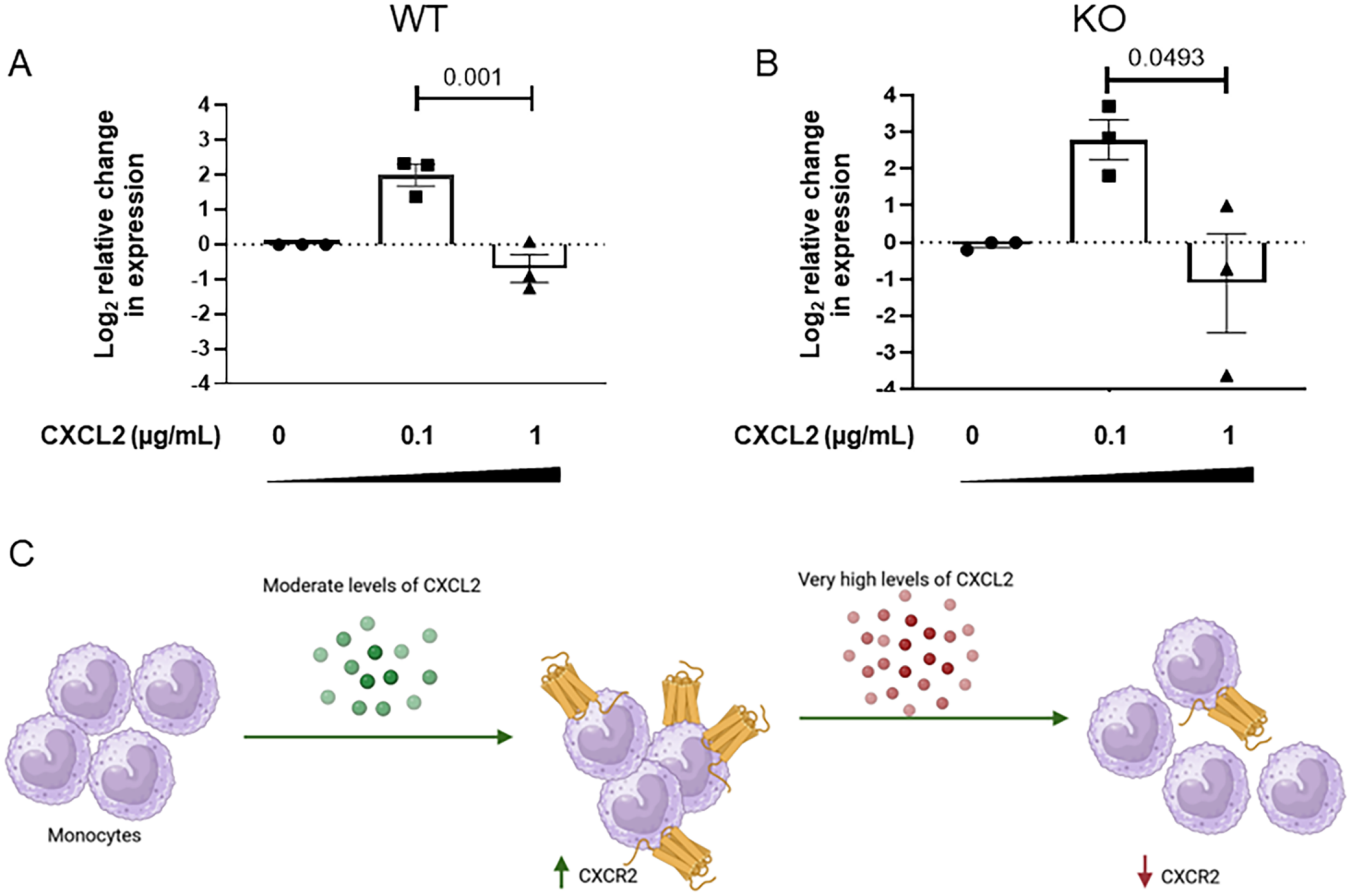
CXCL2 levels regulate CXCR2 receptor expression on neonatal mononuclear cells. Spleens from neonatal WT and KO mice (n=5) were collected on day 7 of life and monocytes were isolated. Cells were stimulated with 0.1 or 1 µg/mL CXCL2 for 8 h Mean gene expression levels of CXCR2 ± SE in **(A)** WT or **(B)** KO mononuclear cells are shown for a combined 3 independent experiments. The expression was determined relative to control by real-time PCR using the formula 2^-ΔΔCt^. Statistical significance was determined using ANOVA; exact P values are shown. **(C)** A schematic to illustrate that lower levels of CXCL2 (green) regulate an increase in CXCR2 expression that is turned off by higher levels of ligand (red).

It is known that overproduction of proinflammatory mediators or persistent exposure to ligand during severe sepsis leads to desensitization of CXCR2 receptor (43). We hypothesized that highly elevated expression levels of CXCL2 chemokine in the WT might be suppressing the expression of chemokine receptor on mononuclear cells during infection. To evaluate this, mononuclear cells were isolated from naïve WT and KO neonatal mice and stimulated with a range of CXCL2 concentrations ex vivo. The results demonstrated that mononuclear cells from both WT and KO pups exhibited elevated expression levels of CXCR2 when stimulated with 0.1 μg/mL of chemokine CXCL2 (**Figures 5A, B**). In contrast, at higher concentration of CXCL2 downregulated the expression of CXCR2 on murine neonatal mononuclear cells (**Figures 5A, B**). This suggests that mononuclear cells from IL-27Ra KO mice are not inherently programmed to express CXCR2 at higher levels than WT, and instead, respond to the environment during infection to modulate their receptor expression level (**Figure 5C**).

### *E. coli*-infected IL-27Rα KO neonates have an expanded population of CXCR2^+^ Ly6C^hi^ monocytes in the spleen

**Figure 6:**
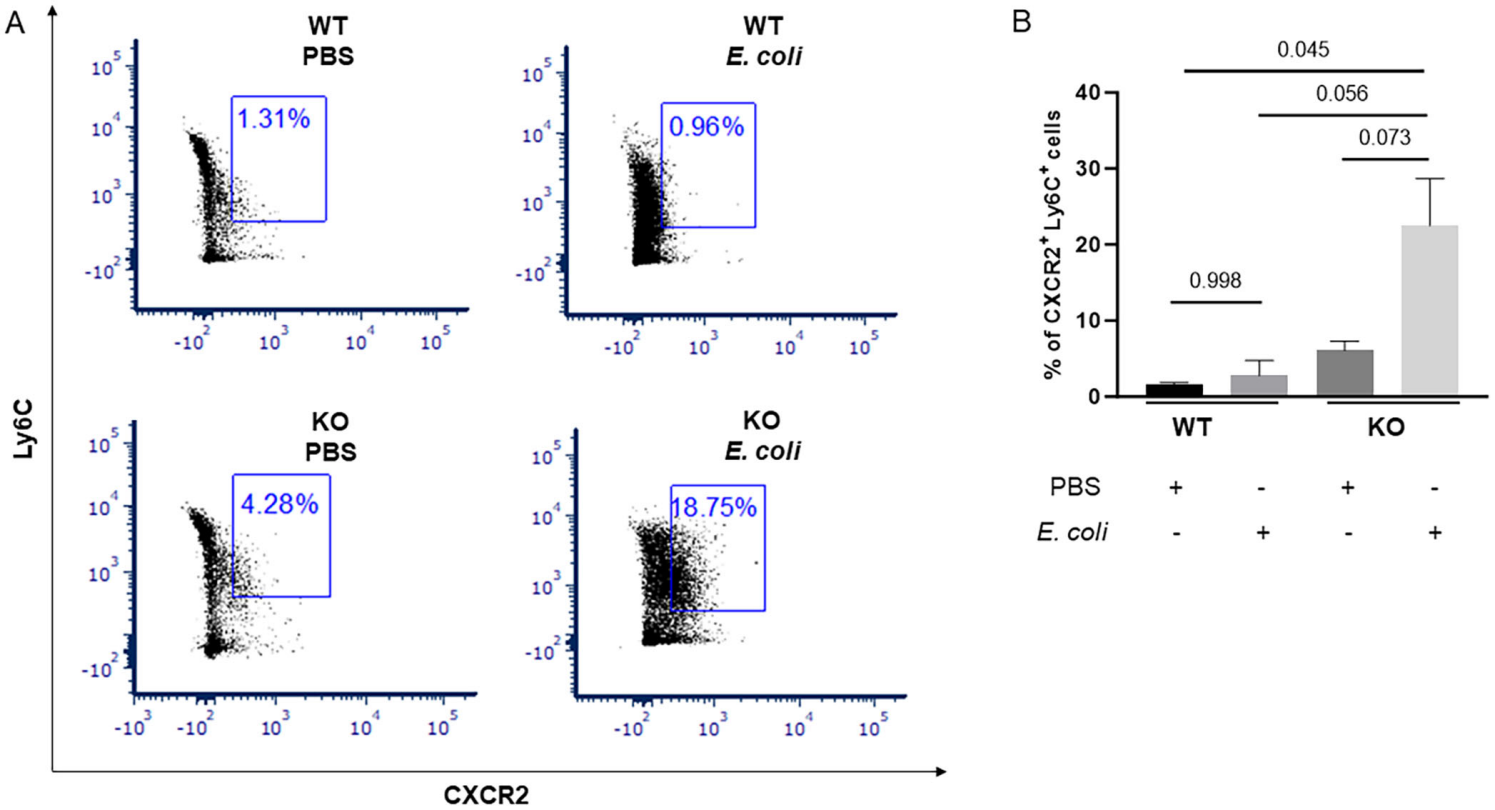
*E. coli*-infected IL-27Ra KO neonates have an expanded population of CXCR2^+^ Ly6C^hi^ mononuclear cells in the spleen. Neonatal WT and KO mice were subcutaneously inoculated with a target of 2×10^5^ CFUs of *E coli* O1:K1:H7 (n=5) or PBS (n=5) as a control on day 4 of life. Spleens were harvested at 24 h post-infection and mononuclear cells isolated by density gradient centrifugation. **(A)** Cells were then stained with antibodies for CD11b, Ly6C, or CXCR2 and analyzed using flow cytometry. The expression of Ly6C and CXCR2 in the single cell live CD11b^+^ cell gate is shown. **(B)** The mean percentage ± SE of Ly6C^+^ CXCR2^+^ cells from a combined 3 independent experiments is shown. Statistical significance was determined using ANOVA; exact P values are shown.

Recruitment of monocytes is essential for effective control and clearance of bacterial infections. Monocytes originate from progenitors in the bone marrow and traffic via the bloodstream to peripheral tissues (44, 45). Monocytes are classified mainly into two subsets with different biological functions based on chemokine receptor expression and the presence of specific surface molecules (46). In mice, classical Ly6C^hi^ monocytes are often referred to as inflammatory monocytes which are rapidly recruited to sites of infection and differentiate further into pro-and anti-inflammatory macrophages which can clear bacteria and promote tissue remodeling or injury (47). To further understand the abundance and phenotype of the monocyte population in the spleen, we isolated mononuclear cells from both WT and KO neonatal mice in the presence and absence of infection at 24 h post-infection for analysis by flow cytometry (**Supplementary Figure 1**). We consistently observed a higher percentage of Ly6C^+^ mononuclear cells that expressed CXCR2 in the infected KO neonates than the WT neonatal pups. (**Figure 6**). These findings mirror the CXCR2 gene expression profiles and suggest enhanced monocyte recruitment to sites of infection.

### Loss of IL-27 signaling reduces tissue injury in the spleen that is consistent with improved morbidity during infection

**Figure 7:**
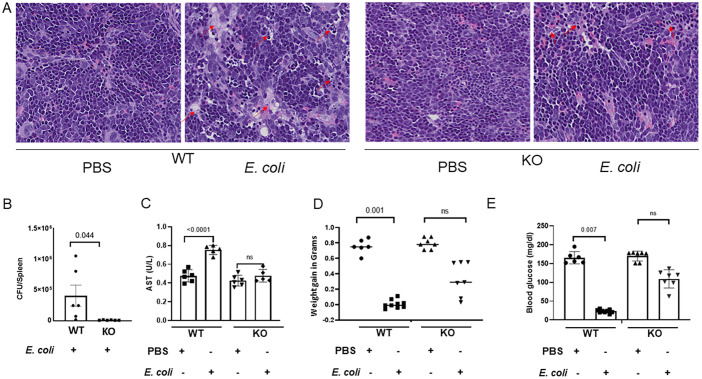
Tissue injury and sepsis-related morbidity is improved in IL-27Rα KO neonates during infection. Neonatal WT and KO mice were subcutaneously inoculated with a target of 2×10^5^ CFUs of *E coli* O1:K1:H7 (n=2-3) or PBS (n=2-3) in each experiment as a control on day 4 of life. Weights were measured at 24 h post-infection immediately prior to collection of blood for glucose or serum analysis and spleens for histopathology. **(A)** Representative H&E-stained sections of spleen from PBS or *E. coli*-inoculated neonatal mice. Red arrows indicate necrotic cells and tissue degeneration. **(B)** Mean bacterial burdens ± SE in the spleen, **(C)** Mean serum AST levels ± SE, and **(D)** mean weights and **(E)** blood glucose values are shown. Each symbol represents an individual mouse from 3 combined experiments. Statistical significance was determined using ANOVA; exact P values are shown.

The spleen is the largest lymphoid tissue and plays an important role in the immune defense to invasive infection, particularly for encapsulated bacteria, and is a key contributor to the exaggerated inflammatory response that occurs during sepsis (48). To explore the potential impact of increased mononuclear cell recruitment in the context of bacterial clearance and tissue injury, we examined spleens harvested at 24 h post-infection from each genotype and treatment condition for bacterial burdens and histopathology. We observed that spleens from infected IL-27Rα KO neonates have well-maintained tissue architecture with minimal damage, in contrast to spleens from the infected WT neonates that demonstrated necrotic regions with shrunken and fragmented cells consistent with increased bacterial burdens (**Figures 7A, B**). Serum ALT and AST levels are a reliable diagnostic tool for sepsis and septic shock that aid in predicting mortality (49). Serum aspartate transaminase (AST) is an enzyme found in the liver, muscles, and other tissues; levels are typically low in the blood and increase following release from damaged cells (50, 51). Elevated levels of AST in the serum correlate with tissue damage or apoptosis, inflammatory liver disease, septic shock, skeletal muscle injury and severe myocardial ischemia (52). A hallmark of sepsis is dysfunction and damage to multiple organs (53, 54), and as such, we measured serum AST levels as an indicator of tissue damage. We found a significant increase in serum AST levels during infection of WT neonates. However, AST levels in KO neonates remained comparable to uninfected control pups (**Figure 7C**). This suggests more limited tissue damage in the KO neonates during infection, in agreement with histological analysis of the spleen. As additional measures of morbidity, we also observed significant weight loss (**Figure 7D**) and hypoglycemia (**Figure 7E**), in the infected WT neonates. Conversely, infected KO neonates demonstrated improved maintenance of weight and blood glucose (**Figures 7D, E**).

## Discussion

neonatal period marks a time of vulnerability and susceptibility to infection with an untrained and suppressive immune environment under adaptation (55). Treatment of neonatal sepsis is a complex clinical challenge as combating both the infection and pathological inflammation is obligatory to improve outcomes (56, 57). Cytokines are important regulators of the immune response, which have a key role in the pathophysiology underlying sepsis (58, 59). Neonates exhibit elevated levels of IL-27, and its signaling compromises control of bacteria (20, 21). In a murine model of neonatal sepsis, mice deficient in IL-27 signaling exhibited reduced mortality, increased weight gain, and better control of bacteria with reduced systemic inflammation (20). The latter is important as strategies to prime or enhance an inflammatory response in neonates to combat the bacterial burden would likely be met with enhanced pathology. Increased clearance of bacteria without inflammation toll suggests a superior formula for bacterial clearance in the absence of IL-27 signaling. This likely involves the regulatory influence of IL-27 on lysosomal activity but may also involve additional undescribed mechanisms (60, 61). Enhanced understanding of the mechanisms by which the absence of IL-27 signaling in neonates promotes resistance to sepsis may lead to development of novel therapeutic approaches. The transcriptome analysis of spleens from *Escherichia coli*-induced septic IL-27Rα-deficient (KO) neonatal mice demonstrated elevated levels of CXCR2 gene expression (25). Here, we further investigated this finding and the impact on immune cell recruitment and the host response during neonatal infection.

CXCR2 plays a central role in the recruitment of circulating neutrophils to sites of inflammation (62). Cummings and colleagues investigated the effect of severe sepsis on the expression and function of the two CXC chemokine receptors on circulating polymorphonuclear neutrophils and found that CXCR2 expression was reduced by 50% in septic patients (63). A separate group reported that down-regulation of CXCR2 on neutrophils prevents migration to the site of infection during severe sepsis (64). We also observed the similar finding of decreased CXCR2 expression levels in the spleen of WT pups during sepsis. Surprisingly, IL-27 receptor KO pups displayed increased levels of CXCR2 in the spleen during sepsis. In contrast to the decreased levels of receptor, its ligand CXCL2 expression was highly upregulated in the WT pups during infection. We demonstrated that purified CXCL2 protein regulated CXCR2 expression in a concentration dependent manner on splenic mononuclear cells. Elevated expression of CXCL2 in the spleens of WT neonates was consistent with reduced expression of CXCR2 on the infected WT splenocytes and consequently their migration towards chemokine *in vitro* was diminished. Conversely, CXCL2 expression was more tightly controlled in the absence of IL-27 signaling, consistent with a higher level of CXCR2 expression. Splenocytes from infected KO pups migrated efficiently towards chemoattractant due to higher CXCR2 receptor expression.

Though the CXCR2 is predominantly expressed on neutrophils, several studies found that upregulation of CXCR2 caused chemotaxis of monocytes and increased monocyte adhesion to endothelial cells (65–67). In our neonatal *E. coli*-induced sepsis mouse model, we found significantly upregulated CXCR2 expression levels in splenic mononuclear cells but not neutrophils of IL-27 receptor KO pups. Neonatal neutrophils differ in their functionality compared to neutrophils of adults. Neutrophils from neonates exhibit a reduced ability to adhere to endothelial surfaces and more limited migration towards chemoattractant resulting in decreased efficiency at combating infections (68, 69). Furthermore, newborns possess a significant population of neutrophils with immune suppressive characteristics known as granulocytic/polymorphonuclear myeloid-derived suppressor cells that inhibit the function of T-cells (GR-MDSC) (70–73). In septic patients, neutrophils can also play a harmful role by facilitating tissue damage and immune-related organ failure; depletion of neutrophils has been shown to significantly reduce lung and liver injury (74). Although neutrophils play a key role in releasing cytokines, as well as phagocytosis and killing of bacteria, dysregulated activity during sepsis further contributes to secondary complications. Conversely, monocytes are central regulators of the inflammatory response and are potential critical elements for the genesis and resolution of sepsis (75). Monocytes from septic patients are modulated/reprogrammed rather than hyporesponsive during sepsis and this modulation may represent the return to homeostasis in cases of successful antimicrobial therapy and recovery of underlying disease (76–78). Monocytes, through rapid differentiation, can expand to macrophages that play essential roles throughout all phases of sepsis and affect both immune homeostasis and inflammatory processes (79, 80). CXCR2^+^ mononuclear cells from the infected KO pups exhibited a CD11b^+^Ly6C^+^ phenotype. Monocytes with this cell surface marker profile have potential to rapidly differentiate into macrophages when they reach tissues. Importantly, we cannot definitively state that all the CD11b^+^Ly6C^+^CXCR2^+^ cells were derived from recruited monocytes. It is likely that many were recruited but some of this population may represent tissue resident cells. Nonetheless, present findings suggest that more efficient recruitment of monocytes may contribute to the explanation of our previous observation of improved bacterial phagocytosis and clearance in the peripheral tissues of infected IL-27Ra KO compared to WT mouse pups (20).

The initial stage of sepsis triggers excessive release of pro-inflammatory cytokines and chemokines, including CXCL2 (81). Several studies have shown that, prolonged or intense exposure to high levels of CXCL2 desensitize and internalize CXCR2 receptors on the surface of neutrophils. This weakens their ability to migrate to the site of infection and eventually impairs pathogen clearance (82–84). In contrast to prior work, in this study we identified CXCR2 receptor desensitization on the mononuclear cells at high concentration of CXCL2. Mononuclear cells from both WT and KO pups exhibited higher expression levels of CXCR2 at lower concentration of CXCL2, whereas higher concentration of CXCL2 downregulated the CXCR2 receptor expression.

**Figure 8:**
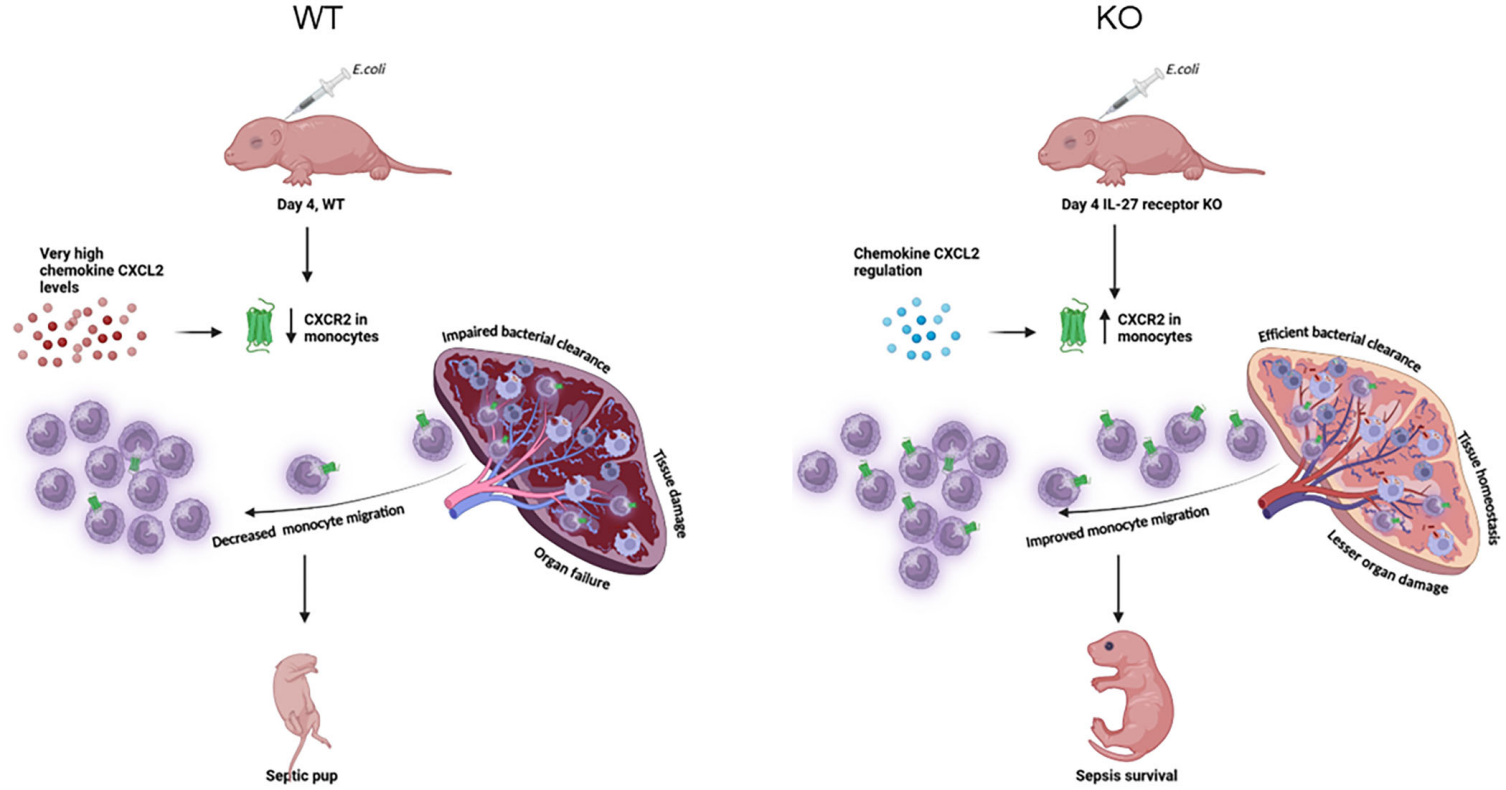
Regulation of immune mechanisms during neonatal sepsis in IL-27Rα KO mice. Inhibition of IL-27 signaling in neonatal mice leads to elevated expression levels of CXCR2 in splenic monocytes due to regulated chemokine levels. Upregulation of CXCR2 subsequently enhances the chemotaxis toward CXCL2. The CXCR2^+^ Ly6C^hi^ mononuclear cells in the spleen promote bacterial clearance and tissue repair in KO neonatal mice during sepsis that ultimately leads to improved survival.

Our future studies aim to determine whether the neutralization of IL-27 cytokine using antibody promotes CXCR2 expression. Limitations of this study include a detailed analysis of the source of CXCL2 in the spleen during infection. Additional single cell studies will be required to understand this thoroughly. Furthermore, a time course kinetic analysis of CXCR2 expression on mononuclear cells and neutrophils in the peripheral blood and bone-marrow was not performed. Consequently, we cannot exclude the onset of changes in CXCR2 expression profiles prior to arrival at the spleen. In conclusion, regulated levels of proinflammatory cytokine (20) and chemokine during *E. coli* infection in IL-27 receptor KO neonatal mice, retains CXCR2 expression on the splenic monocyte which causes effective migration to the site of infection and is consistent with effective bacterial clearance and tissue homeostasis. Limiting IL-27 signaling in neonates has the potential to balance the immune response during infection with maximum bacterial clearance, minimal tissue damage and improved survival (**Figure 8**).

## Data Availability

The datasets presented in this study can be found in online repositories. The names of the repository/repositories and accession number(s) can be found below: https://www.ncbi.nlm.nih.gov/geo/, GSE220050.

## References

[1] Vincent JL. Sepsis and infection: Two words that should not be confused. Front Med (Lausanne). (2023) 10:1156732. doi: 10.3389/fmed.2023.1156732, PMID: 36968843 PMC10033658

[2] Milton R Gillespie D Dyer C Taiyari K Carvalho MJ Thomson K. Neonatal sepsis and mortality in low-income and middle-income countries from a facility-based birth cohort: an international multisite prospective observational study. Lancet Glob Health. (2022) 10:e661–e72. doi: 10.1016/S2214-109X(22)00043-2, PMID: 35427523 PMC9023753

[3] Stoll BJ Puopolo KM Hansen NI Sanchez PJ Bell EF Carlo WA. Early-onset neonatal sepsis 2015 to 2017, the rise of escherichia coli, and the need for novel prevention strategies. JAMA Pediatr. (2020) 174:e200593. doi: 10.1001/jamapediatrics.2020.0593, PMID: 32364598 PMC7199167

[4] Yadav P Yadav SK. Progress in diagnosis and treatment of neonatal sepsis: A review article. JNMA J Nepal Med Assoc. (2022) 60:318–24. doi: 10.31729/jnma.7324, PMID: 35633256 PMC9226748

[5] Flannery DD Edwards EM Coggins SA Horbar JD Puopolo KM. Late-onset sepsis among very preterm infants. Pediatrics. (2022) 150:e2022058813. doi: 10.1542/peds.2022-058813, PMID: 36366916 PMC11151779

[6] Guideline: managing possible serious bacterial infection in young infants when referral is not feasible. Geneva: WHO Guidelines Approved by the Guidelines Review Committee (2015). 26447263

[7] Neonatal infection: antibiotics for prevention and treatment. London: National Institute for Health and Care Excellence: Guidelines (2024). 34133110

[8] Korang SK Safi S Gluud C Lausten-Thomsen U Jakobsen JC. Antibiotic regimens for neonatal sepsis - a protocol for a systematic review with meta-analysis. Syst Rev. (2019) 8:306. doi: 10.1186/s13643-019-1207-1, PMID: 31805993 PMC6896287

[9] Sokou R Parastatidou S Konstantinidi A Tsantes AG Iacovidou N. Editorial: Neonatal sepsis: current insights and challenges. Front Pediatr. (2024) 12:1427503. doi: 10.3389/fped.2024.1427503, PMID: 38868391 PMC11167085

[10] Carbone F Montecucco F Sahebkar A. Current and emerging treatments for neonatal sepsis. Expert Opin Pharmacother. (2020) 21:549–56. doi: 10.1080/14656566.2020.1721464, PMID: 32011188

[11] Tsafaras GP Ntontsi P Xanthou G. Advantages and limitations of the neonatal immune system. Front Pediatr. (2020) 8:5. doi: 10.3389/fped.2020.00005, PMID: 32047730 PMC6997472

[12] Kollmann TR Kampmann B Mazmanian SK Marchant A Levy O. Protecting the newborn and young infant from infectious diseases: lessons from immune ontogeny. Immunity. (2017) 46:350–63. doi: 10.1016/j.immuni.2017.03.009, PMID: 28329702

[13] Pflanz S Timans JC Cheung J Rosales R Kanzler H Gilbert J. IL-27, a heterodimeric cytokine composed of EBI3 and p28 protein, induces proliferation of naive CD4+ T cells. Immunity. (2002) 16:779–90. doi: 10.1016/s1074-7613(02)00324-2, PMID: 12121660

[14] Collison LW Workman CJ Kuo TT Boyd K Wang Y Vignali KM. The inhibitory cytokine IL-35 contributes to regulatory T-cell function. Nature. (2007) 450:566–9. doi: 10.1038/nature06306, PMID: 18033300

[15] Ziblat A Domaica CI Spallanzani RG Iraolagoitia XL Rossi LE Avila DE. IL-27 stimulates human NK-cell effector functions and primes NK cells for IL-18 responsiveness. Eur J Immunol. (2015) 45:192–202. doi: 10.1002/eji.201444699, PMID: 25308526

[16] Xu WD Wang DC Zhao M Huang AF. An updated advancement of bifunctional IL-27 in inflammatory autoimmune diseases. Front Immunol. (2024) 15:1366377. doi: 10.3389/fimmu.2024.1366377, PMID: 38566992 PMC10985211

[17] Yoshida H Hunter CA. The immunobiology of interleukin-27. Annu Rev Immunol. (2015) 33:417–43. doi: 10.1146/annurev-immunol-032414-112134, PMID: 25861977

[18] Klarquist J Cross EW Thompson SB Willett B Aldridge DL Caffrey-Carr AK. B cells promote CD8 T cell primary and memory responses to subunit vaccines. Cell Rep. (2021) 36:109591. doi: 10.1016/j.celrep.2021.109591, PMID: 34433030 PMC8456706

[19] Wong HR Liu KD Kangelaris KN Lahni P Calfee CS. Performance of interleukin-27 as a sepsis diagnostic biomarker in critically ill adults. J Crit Care. (2014) 29:718–22. doi: 10.1016/j.jcrc.2014.04.004, PMID: 24848949 PMC4141017

[20] Seman BG Vance JK Rawson TW Witt MR Huckaby AB Povroznik JM. Elevated levels of interleukin-27 in early life compromise protective immunity in a mouse model of gram-negative neonatal sepsis. Infect Immun. (2020) 88:e00828-19. doi: 10.1128/IAI.00828-19, PMID: 31818960 PMC7035946

[21] Kraft JD Horzempa J Davis C Jung JY Pena MM Robinson CM. Neonatal macrophages express elevated levels of interleukin-27 that oppose immune responses. Immunology. (2013) 139:484–93. doi: 10.1111/imm.12095, PMID: 23464355 PMC3719065

[22] Jung JY Gleave Parson M Kraft JD Lyda L Kobe B Davis C. Elevated interleukin-27 levels in human neonatal macrophages regulate indoleamine dioxygenase in a STAT-1 and STAT-3-dependent manner. Immunology. (2016) 149:35–47. doi: 10.1111/imm.12625, PMID: 27238498 PMC4981608

[23] Kelly AM Leech JM Doyle SL McLoughlin RM. Staphylococcus aureus-induced immunosuppression mediated by IL-10 and IL-27 facilitates nasal colonisation. PloS Pathog. (2022) 18:e1010647. doi: 10.1371/journal.ppat.1010647, PMID: 35776778 PMC9282462

[24] Xu F Liu Q Lin S Shen N Yin Y Cao J. IL-27 is elevated in acute lung injury and mediates inflammation. J Clin Immunol. (2013) 33:1257–68. doi: 10.1007/s10875-013-9923-0, PMID: 23842867 PMC7102048

[25] Povroznik JM Akhter H Vance JK Annamanedi M Dziadowicz SA Wang L. Interleukin-27-dependent transcriptome signatures during neonatal sepsis. Front Immunol. (2023) 14:1124140. doi: 10.3389/fimmu.2023.1124140, PMID: 36891292 PMC9986606

[26] Lazennec G Rajarathnam K Richmond A. CXCR2 chemokine receptor - a master regulator in cancer and physiology. Trends Mol Med. (2024) 30:37–55. doi: 10.1016/j.molmed.2023.09.003, PMID: 37872025 PMC10841707

[27] Eash KJ Greenbaum AM Gopalan PK Link DC. CXCR2 and CXCR4 antagonistically regulate neutrophil trafficking from murine bone marrow. J Clin Invest. (2010) 120:2423–31. doi: 10.1172/JCI41649, PMID: 20516641 PMC2898597

[28] Zhang YL Cao HJ Han X Teng F Chen C Yang J. Chemokine receptor CXCR-2 initiates atrial fibrillation by triggering monocyte mobilization in mice. Hypertension. (2020) 76:381–92. doi: 10.1161/HYPERTENSIONAHA.120.14698, PMID: 32639881

[29] Kaczmarek A Budzynska A Gospodarek E. Detection of K1 antigen of Escherichia coli rods isolated from pregnant women and neonates. Folia Microbiol (Praha). (2014) 59:419–22. doi: 10.1007/s12223-014-0315-5, PMID: 24737297 PMC4133638

[30] Justus CR Leffler N Ruiz-Echevarria M Yang LV. *In vitro* cell migration and invasion assays. J Vis Exp. (2014) 88:51046. doi: 10.3791/51046, PMID: 24962652 PMC4186330

[31] Robinson CM Nau GJ. Interleukin-12 and interleukin-27 regulate macrophage control of Mycobacterium tuberculosis. J Infect Dis. (2008) 198:359–66. doi: 10.1086/589774, PMID: 18557702 PMC2761687

[32] Seman BG Povroznik JM Vance JK Rawson TW Robinson CM. A neonatal imaging model of gram-negative bacterial sepsis. J Vis Exp. (2020) 162. doi: 10.3791/61609, PMID: 32865536

[33] Boon K Vanalken N Szpakowska M Chevigne A Schols D Van Loy T. Systematic assessment of chemokine ligand bias at the human chemokine receptor CXCR2 indicates G protein bias over beta-arrestin recruitment and receptor internalization. Cell Commun Signal. (2024) 22:43. doi: 10.1186/s12964-023-01460-2, PMID: 38233929 PMC10795402

[34] Lewis SM Williams A Eisenbarth SC. Structure and function of the immune system in the spleen. Sci Immunol. (2019) 4:eaau6085. doi: 10.1126/sciimmunol.aau6085, PMID: 30824527 PMC6495537

[35] Bronte V Pittet MJ. The spleen in local and systemic regulation of immunity. Immunity. (2013) 39:806–18. doi: 10.1016/j.immuni.2013.10.010, PMID: 24238338 PMC3912742

[36] Chapman RW Phillips JE Hipkin RW Curran AK Lundell D Fine JS. CXCR2 antagonists for the treatment of pulmonary disease. Pharmacol Ther. (2009) 121:55–68. doi: 10.1016/j.pharmthera.2008.10.005, PMID: 19026683

[37] Matsukawa A Hogaboam CM Lukacs NW Kunkel SL. Chemokines and innate immunity. Rev Immunogenet. (2000) 2:339–58. 11256744

[38] Navarro-Xavier RA Newson J Silveira VL Farrow SN Gilroy DW Bystrom J. A new strategy for the identification of novel molecules with targeted proresolution of inflammation properties. J Immunol. (2010) 184:1516–25. doi: 10.4049/jimmunol.0902866, PMID: 20032295

[39] Rot A McKimmie C Burt CL Pallas KJ Jamieson T Pruenster M. Cell-autonomous regulation of neutrophil migration by the D6 chemokine decoy receptor. J Immunol. (2013) 190:6450–6. doi: 10.4049/jimmunol.1201429, PMID: 23670187 PMC4821389

[40] Wang H Shao Q Wang J Zhao L Wang L Cheng Z. Decreased CXCR2 expression on circulating monocytes of colorectal cancer impairs recruitment and induces Re-education of tumor-associated macrophages. Cancer Lett. (2022) 529:112–25. doi: 10.1016/j.canlet.2022.01.004, PMID: 34999169

[41] O’Shea TM Wollenberg AL Kim JH Ao Y Deming TJ Sofroniew MV. Foreign body responses in mouse central nervous system mimic natural wound responses and alter biomaterial functions. Nat Commun. (2020) 11:6203. doi: 10.1038/s41467-020-19906-3, PMID: 33277474 PMC7718896

[42] Seman BG Vance JK Akers SM Robinson CM. Neonatal low-density granulocytes internalize and kill bacteria but suppress monocyte function using extracellular DNA. J Cell Sci. (2021) 134:jcs252528. doi: 10.1242/jcs.252528, PMID: 33589502

[43] Korbecki J Kupnicka P Chlubek M Goracy J Gutowska I Baranowska-Bosiacka I. CXCR2 receptor: regulation of expression, signal transduction, and involvement in cancer. Int J Mol Sci. (2022) 23:2168. doi: 10.3390/ijms23042168, PMID: 35216283 PMC8878198

[44] Auffray C Sieweke MH Geissmann F. Blood monocytes: development, heterogeneity, and relationship with dendritic cells. Annu Rev Immunol. (2009) 27:669–92. doi: 10.1146/annurev.immunol.021908.132557, PMID: 19132917

[45] van Furth R Cohn ZA. The origin and kinetics of mononuclear phagocytes. J Exp Med. (1968) 128:415–35. doi: 10.1084/jem.128.3.415, PMID: 5666958 PMC2138527

[46] Geissmann F Jung S Littman DR. Blood monocytes consist of two principal subsets with distinct migratory properties. Immunity. (2003) 19:71–82. doi: 10.1016/s1074-7613(03)00174-2, PMID: 12871640

[47] Serbina NV Jia T Hohl TM Pamer EG. Monocyte-mediated defense against microbial pathogens. Annu Rev Immunol. (2008) 26:421–52. doi: 10.1146/annurev.immunol.26.021607.090326, PMID: 18303997 PMC2921669

[48] Chen H Huang N Tian H Li J Li B Sun J. Splenectomy provides protective effects against CLP-induced sepsis by reducing TRegs and PD-1/PD-L1 expression. Int J Biochem Cell Biol. (2021) 136:105970. doi: 10.1016/j.biocel.2021.105970, PMID: 33774183

[49] Schupp T Weidner K Rusnak J Jawhar S Forner J Dulatahu F. Diagnostic and prognostic value of the AST/ALT ratio in patients with sepsis and septic shock. Scand J Gastroenterol. (2023) 58:392–402. doi: 10.1080/00365521.2022.2131331, PMID: 36259154

[50] Panteghini M. Aspartate aminotransferase isoenzymes. Clin Biochem. (1990) 23:311–9. doi: 10.1016/0009-9120(90)80062-N, PMID: 2225456

[51] Yang RZ Park S Reagan WJ Goldstein R Zhong S Lawton M. Alanine aminotransferase isoenzymes: molecular cloning and quantitative analysis of tissue expression in rats and serum elevation in liver toxicity. Hepatology. (2009) 49:598–607. doi: 10.1002/hep.22657, PMID: 19085960 PMC2917112

[52] Ndrepepa G. Aspartate aminotransferase and cardiovascular disease—a narrative review. J Lab Precis Med. (2020) 6:6. doi: 10.21037/jlpm-20-93

[53] Lappin E Ferguson AJ. Gram-positive toxic shock syndromes. Lancet Infect Dis. (2009) 9:281–90. doi: 10.1016/S1473-3099(09)70066-0, PMID: 19393958

[54] Priyanka S Morkar D. AST/ALT Ratio as an indicator of functional severity in chronic heart failure with reduced left ventricular ejection fraction: A prospective cross-sectional study. Indian Heart J. (2024) 76:202–6. doi: 10.1016/j.ihj.2024.06.004, PMID: 38897408 PMC11329059

[55] Basha S Surendran N Pichichero M. Immune responses in neonates. Expert Rev Clin Immunol. (2014) 10:1171–84. doi: 10.1586/1744666X.2014.942288, PMID: 25088080 PMC4407563

[56] Zea-Vera A Ochoa TJ. Challenges in the diagnosis and management of neonatal sepsis. J Trop Pediatr. (2015) 61:1–13. doi: 10.1093/tropej/fmu079, PMID: 25604489 PMC4375388

[57] MaChado JR Soave DF da Silva MV de Menezes LB Etchebehere RM Monteiro ML. Neonatal sepsis and inflammatory mediators. Mediators Inflamm. (2014) 2014:269681. doi: 10.1155/2014/269681, PMID: 25614712 PMC4295603

[58] Wu J Xie A Chen W. Cytokine regulation of immune tolerance. Burns Trauma. (2014) 2:11–7. doi: 10.4103/2321-3868.124771, PMID: 27574641 PMC4994505

[59] Chaudhry H Zhou J Zhong Y Ali MM McGuire F Nagarkatti PS. Role of cytokines as a double-edged sword in sepsis. In Vivo. (2013) 27:669–84. PMID: 24292568 PMC4378830

[60] Jung JY Robinson CM. Interleukin-27 inhibits phagosomal acidification by blocking vacuolar ATPases. Cytokine. (2013) 62:202–5. doi: 10.1016/j.cyto.2013.03.010, PMID: 23557795 PMC3760007

[61] Jung JY Robinson CM. IL-12 and IL-27 regulate the phagolysosomal pathway in mycobacteria-infected human macrophages. Cell Commun Signal. (2014) 12:16. doi: 10.1186/1478-811X-12-16, PMID: 24618498 PMC4007735

[62] Olson TS Ley K. Chemokines and chemokine receptors in leukocyte trafficking. Am J Physiol Regul Integr Comp Physiol. (2002) 283:R7–28. doi: 10.1152/ajpregu.00738.2001, PMID: 12069927

[63] Cummings CJ Martin TR Frevert CW Quan JM Wong VA Mongovin SM. Expression and function of the chemokine receptors CXCR1 and CXCR2 in sepsis. J Immunol. (1999) 162:2341–6. doi: 10.4049/jimmunol.162.4.2341, PMID: 9973513

[64] Rios-Santos F Alves-Filho JC Souto FO Spiller F Freitas A Lotufo CM. Down-regulation of CXCR2 on neutrophils in severe sepsis is mediated by inducible nitric oxide synthase-derived nitric oxide. Am J Respir Crit Care Med. (2007) 175:490–7. doi: 10.1164/rccm.200601-103OC, PMID: 17138957

[65] Lei ZB Zhang Z Jing Q Qin YW Pei G Cao BZ. OxLDL upregulates CXCR2 expression in monocytes via scavenger receptors and activation of p38 mitogen-activated protein kinase. Cardiovasc Res. (2002) 53:524–32. doi: 10.1016/S0008-6363(01)00491-6, PMID: 11827704

[66] Han X Shi H Sun Y Shang C Luan T Wang D. CXCR2 expression on granulocyte and macrophage progenitors under tumor conditions contributes to mo-MDSC generation via SAP18/ERK/STAT3. Cell Death Dis. (2019) 10:598. doi: 10.1038/s41419-019-1837-1, PMID: 31395859 PMC6687752

[67] Dyer DP Pallas K Medina-Ruiz L Schuette F Wilson GJ Graham GJ. CXCR2 deficient mice display macrophage-dependent exaggerated acute inflammatory responses. Sci Rep. (2017) 7:42681. doi: 10.1038/srep42681, PMID: 28205614 PMC5311995

[68] Lawrence SM Corriden R Nizet V. Age-appropriate functions and dysfunctions of the neonatal neutrophil. Front Pediatr. (2017) 5:23. doi: 10.3389/fped.2017.00023, PMID: 28293548 PMC5329040

[69] Fox SE Lu W Maheshwari A Christensen RD Calhoun DA. The effects and comparative differences of neutrophil specific chemokines on neutrophil chemotaxis of the neonate. Cytokine. (2005) 29:135–40. doi: 10.1016/j.cyto.2004.10.007, PMID: 15613281

[70] Schwarz J Scheckenbach V Kugel H Spring B Pagel J Hartel C. Granulocytic myeloid-derived suppressor cells (GR-MDSC) accumulate in cord blood of preterm infants and remain elevated during the neonatal period. Clin Exp Immunol. (2018) 191:328–37. doi: 10.1111/cei.13059, PMID: 28963753 PMC5801499

[71] Gervassi A Lejarcegui N Dross S Jacobson A Itaya G Kidzeru E. Myeloid derived suppressor cells are present at high frequency in neonates and suppress *in vitro* T cell responses. PloS One. (2014) 9:e107816. doi: 10.1371/journal.pone.0107816, PMID: 25248150 PMC4172591

[72] Vance JK Rawson TW Povroznik JM Brundage KM Robinson CM. Myeloid-derived suppressor cells gain suppressive function during neonatal bacterial sepsis. Int J Mol Sci. (2021) 22:7047. doi: 10.3390/ijms22137047, PMID: 34208904 PMC8268718

[73] Gleave Parson M Grimmett J Vance JK Witt MR Seman BG Rawson TW. Murine myeloid-derived suppressor cells are a source of elevated levels of interleukin-27 in early life and compromise control of bacterial infection. Immunol Cell Biol. (2019) 97:445–56. doi: 10.1111/imcb.12224, PMID: 30575117 PMC6536317

[74] Wickel DJ Cheadle WG Mercer-Jones MA Garrison RN. Poor outcome from peritonitis is caused by disease acuity and organ failure, not recurrent peritoneal infection. Ann Surg. (1997) 225:744–53. doi: 10.1097/00000658-199706000-00012, PMID: 9230815 PMC1190882

[75] de Azambuja Rodrigues PM Valente RH Brunoro GVF Nakaya HTI Araujo-Pereira M Bozza PT. Proteomics reveals disturbances in the immune response and energy metabolism of monocytes from patients with septic shock. Sci Rep. (2021) 11:15149. doi: 10.1038/s41598-021-94474-0, PMID: 34312428 PMC8313678

[76] Salomao R Brunialti MK Rapozo MM Baggio-Zappia GL Galanos C Freudenberg M. Bacterial sensing, cell signaling, and modulation of the immune response during sepsis. Shock. (2012) 38:227–42. doi: 10.1097/SHK.0b013e318262c4b0, PMID: 22777111

[77] Cavaillon JM Adib-Conquy M. Bench-to-bedside review: endotoxin tolerance as a model of leukocyte reprogramming in sepsis. Crit Care. (2006) 10:233. doi: 10.1186/cc5055, PMID: 17044947 PMC1751079

[78] Santos SS Carmo AM Brunialti MK MaChado FR Azevedo LC Assuncao M. Modulation of monocytes in septic patients: preserved phagocytic activity, increased ROS and NO generation, and decreased production of inflammatory cytokines. Intensive Care Med Exp. (2016) 4:5. doi: 10.1186/s40635-016-0078-1, PMID: 26879814 PMC4754229

[79] Cheng Y Marion TN Cao X Wang W Cao Y. Park 7: A novel therapeutic target for macrophages in sepsis-induced immunosuppression. Front Immunol. (2018) 9:2632. doi: 10.3389/fimmu.2018.02632, PMID: 30542343 PMC6277877

[80] Surbatovic M Veljovic M Jevdjic J Popovic N Djordjevic D Radakovic S. Immunoinflammatory response in critically ill patients: severe sepsis and/or trauma. Mediators Inflamm. (2013) 2013:362793. doi: 10.1155/2013/362793, PMID: 24371374 PMC3859159

[81] Doganyigit Z Eroglu E Akyuz E. Inflammatory mediators of cytokines and chemokines in sepsis: From bench to bedside. Hum Exp Toxicol. (2022) 41:9603271221078871. doi: 10.1177/09603271221078871, PMID: 35337213

[82] Mueller SG White JR Schraw WP Lam V Richmond A. Ligand-induced desensitization of the human CXC chemokine receptor-2 is modulated by multiple serine residues in the carboxyl-terminal domain of the receptor. J Biol Chem. (1997) 272:8207–14. doi: 10.1074/jbc.272.13.8207, PMID: 9079638

[83] Liu N Bauer M Press AT. The immunological function of CXCR2 in the liver during sepsis. J Inflammation (Lond). (2022) 19:23. doi: 10.1186/s12950-022-00321-y, PMID: 36451225 PMC9714102

[84] Ness TL Hogaboam CM Strieter RM Kunkel SL. Immunomodulatory role of CXCR2 during experimental septic peritonitis. J Immunol. (2003) 171:3775–84. doi: 10.4049/jimmunol.171.7.3775, PMID: 14500678

