# Supplementary material for: Inhibition of IL-27 signaling regulates chemokine levels and sustains CXCR2 receptor expression on mononuclear cells to improve disease outcomes during gram-negative neonatal sepsis
Source: Front Immunol. 2025 Sep 5;16:1653355. doi: 10.3389/fimmu.2025.1653355 (PMC12446255; doi:10.3389/fimmu.2025.1653355)
Supplement: Supplementary file 1 [file Supplementaryfile1.docx]

**
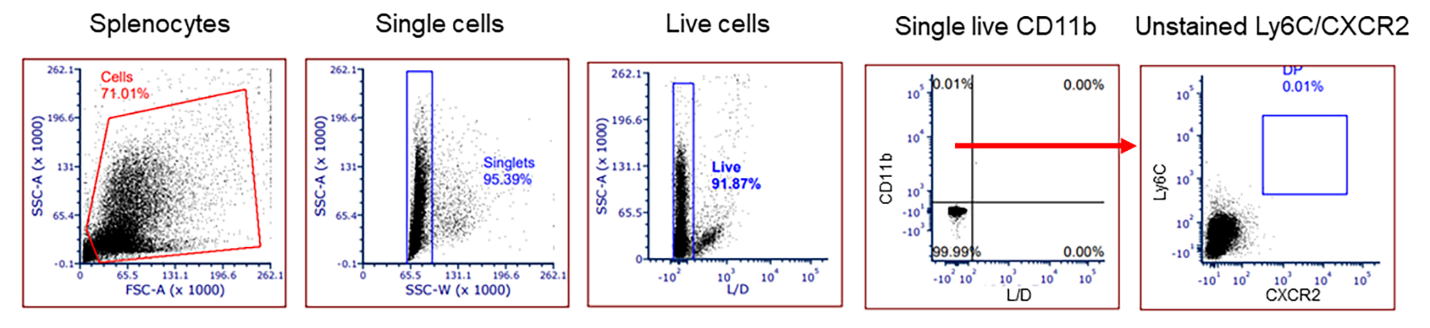
**

**Supplementary figure 1. Gating strategy for flow-cytometry-based sorting of CXCR2^+^ Ly6C^hi^** **cells**. The plots show sequentially the gating hierarchy of one representative sample: Splenocytes, single cells, live cells. The live CD11b+ cell population was gated for Ly6C and CXCR2 labeling. Unstained splenocytes are used to determine the background autofluorescence to set the negative population.
